# Circ*MTA2* Drives Gastric Cancer Progression through Suppressing *MTA2* Degradation via Interacting with UCHL3

**DOI:** 10.3390/ijms25052817

**Published:** 2024-02-29

**Authors:** Gengchen Xie, Bo Lei, Zhijie Yin, Fei Xu, Xinghua Liu

**Affiliations:** Department of Gastrointestinal Surgery, Union Hospital, Tongji Medical College, Huazhong University of Science and Technology, Wuhan 430022, China; gengchen_xie@hust.edu.cn (G.X.); d201478231@alumni.hust.edu.cn (B.L.); jad@hust.edu.cn (Z.Y.)

**Keywords:** CircRNAs, *MTA2*, gastric cancer, UCHL3, ubiquitination

## Abstract

Our previous study has reported that metastasis-associated protein 2 (*MTA2*) plays essential roles in tumorigenesis and aggressiveness of gastric cancer (GC). However, the underlying molecular mechanisms of *MTA2*-mediated GC and its upstream regulation mechanism remain elusive. In this study, we identified a novel circular RNA (circRNA) generated from the *MTA2* gene (circ*MTA2*) as a crucial regulator in GC progression. Circ*MTA2* was highly expressed in GC tissues and cell lines, and circ*MTA2* promoted the proliferation, invasion, and metastasis of GC cells both in vitro and in vivo. Mechanistically, circ*MTA2* interacted with ubiquitin carboxyl-terminal hydrolase L3 (UCHL3) to restrain *MTA2* ubiquitination and stabilize *MTA2* protein expression, thereby facilitating tumor progression. Moreover, circ*MTA2* was mainly encapsulated and transported by exosomes to promote GC cell progression. Taken together, these findings uncover that circ*MTA2* suppresses *MTA2* degradation by interacting with UCHL3, thereby promoting GC progression. In conclusion, we identified a cancer-promoting axis (circ*MTA2*/UCHL3/*MTA2*) in GC progression, which paves the way for us to design and synthesize targeted inhibitors as well as combination therapies.

## 1. Introduction

Gastric cancer (GC) is one of the most commonly diagnosed malignant tumors and the fourth leading cause of cancer-related death worldwide [1,2]. Despite advances in diagnostic methods and surgical therapies, the overall survival rate for patients diagnosed with GC is still dismal in recent years [3]. Given the genetic and epigenetic alterations within tumor tissues, the treatment of GC is very complicated, and the 5-year overall survival rate is currently less than 30% in most countries [4,5]. The issue of the economic burden of colorectal cancer persists and requires efforts from multiple aspects to address it, including strengthening early screening and prevention measures, promoting effective treatment methods, and reducing medical costs. At the same time, more resources and funding should be allocated to research and develop innovative treatment methods to reduce treatment costs and improve treatment outcomes. Therefore, comprehending the molecular mechanisms of GC occurrence and development is urgently needed.

Circular RNAs (circRNAs) are a new class of endogenous non-coding RNAs (ncRNAs), which are generated by precursor mRNA back splicing and form a covalently closed circular structure [6]. Owing to this unique structure, circRNAs have a longer half-life and more resistance to RNase R than linear RNAs, making them recognizable as promising biomarkers and targets for diagnosing and treating human diseases, especially cancers [6,7]. Increasing evidence suggests that the ectopic expression of circRNAs plays a fundamental regulatory role in the occurrence and development of various tumors [7,8]. Recently, plenty of studies have uncovered the diverse functional roles of circRNAs, such as regulating transcription of parental genes in the nucleus, sponging of miRNAs and RNA-binding proteins (RBPs), and regulation of downstream gene translation in the cytoplasm [9,10,11]. For example, circMBOAT2 was found to promote pancreatic cancer progression and glutamine catabolism by acting as a miR-433-3p sponge [9]. In breast cancer, circRNF20 could harbor miR-487a to regulate hypoxia-inducible factor-1α (HIF-1α) expression and subsequently promote proliferation and the Warburg effect of tumor cells [10]. In addition, circHuR interacted with CCHC-type zinc finger nucleic acid binding protein (CNBP) and subsequently restrained HuR expression, thereby resulting in the repression of GC progression [11]. Currently, the expression landscapes of circRNA in GC and the comprehensive link between circRNAs and RBPs remain largely unknown and require further investigation.

Metastasis tumor-associated 2 (*MTA2*), as a member of the MTA family, plays a vital role in modulating gene expression [12]. Studies have demonstrated that *MTA2* is highly expressed in many types of cancers and is associated with tumor formation and progression [13,14]. Previously, we found that elevated *MTA2* levels were associated with short overall survival and disease-free survival in GC specimens [15]. Moreover, *MTA2* could bind with the promoter of MCM5 and promote its transcription, thereby regulating GC progression [15]. However, the upstream regulators of *MTA2* in the progression of GC cells remain poorly understood.

In the present study, we identified a previously unknown circRNA named circ*MTA2* (hsa_circ_0022462) derived from the *MTA2* gene, expanding our knowledge of the *MTA2* gene’s transcriptional landscape and uncovering the functional role of circ*MTA2* in promoting proliferation, invasion, and metastasis of GC cells both in vitro and in vivo. T Mechanistically, circ*MTA2* was found to suppress *MTA2* ubiquitination and stabilize *MTA2* protein expression via interacting with ubiquitin carboxyl-terminal hydrolase L3 (UCHL3). In general, our study reveals an essential role of the circ*MTA2*/UCHL3/*MTA2* axis in GC progression, expanding the current knowledge on the molecular mechanism of *MTA2*-mediated cancer progress.

## 2. Results

### 2.1. CircMTA2 Is Upregulated in the GC Tissue and Cells

**Figure 1:**
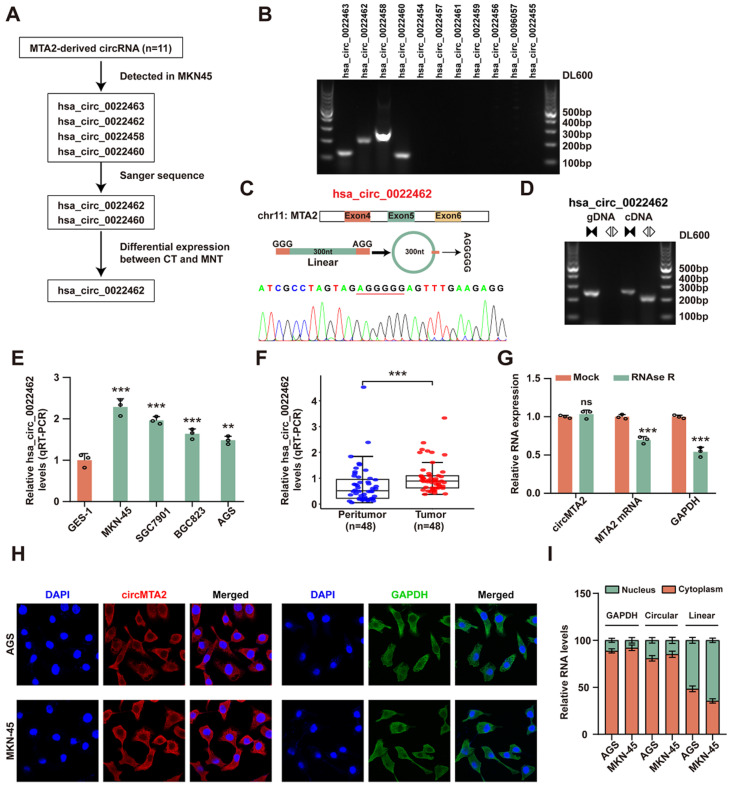
Circ*MTA2* has higher expression in the GC tissue and cells. (**A**) circ*MTA2* screening flowchart. (**B**) The expression of 11 screened *MTA2*-derived circRNAs in AGS cells. (**C**) Genomic location and Sanger sequence validation of circ*MTA2*. (**D**) qRT-PCR assay indicating the circularization structure of circ*MTA2*. (**E**) Relative hsa_circ_0022462 expression levels in GC and GES-1 cells. (**F**) Relative hsa_circ_0022462 expression levels in GC tissues and adjacent normal tissues. (**G**) qRT-PCR assay indicating the expression of circ*MTA2* and *MTA2* mRNA in AGS cells administered with RNase R or Mock control. (**H**) RNA FISH assay showing the localization of circ*MTA2* in AGS and MKN-45 cells. GAPDH was applied as a positive control; Scale: 100×. (**I**) The subcellular location analysis reveals the distribution of circ*MTA2* in the cytoplasm and nucleus. All data from three independent experiments shown as mean ± SD. ns *p* > 0.05, ** *p* < 0.01; *** *p* < 0.001.

First, we explored the expression profile of circRNAs derived from the *MTA2* gene in the GC cell line (Figure 1A). As shown in Figure 1B, only 4 of 11 candidate circRNAs were detected in MKN45 cells. We further conducted Sanger sequencing on the PCR products of these four circRNAs and found that only hsa_circ_0022462 and hsa_circ_0022460 were consistent with the sequence information in the circBase database. Hsa_circ_0022462 is derived from the *MTA2* gene exon 4, 5, and 6, with a spliced mature sequence length of 300 nt (Figure 1C). Hsa_circ_0022460 is derived from the *MTA2* gene and comprises the head-to-tail splicing of exon 13 (140 nt) (Figure S1A). Moreover, the back splicing junctions of hsa_circ_0022462 and hsa_circ_0022460 were amplified using divergent primers only in cDNA samples but not in genomic DNA samples (Figure 1D and Figure S1B). Subsequently, the expression levels of hsa_circ_0022462 and hsa_circ_0022460 were investigated in GC cell lines and tissues. Hsa_circ_0022462 was significantly upregulated in GC cell lines (MKN-45, SGC7901, BGC823, and AGS) compared with normal human gastric mucosa epithelial cells (GES-1) (Figure 1E). Similar results were also observed in GC tissue (Tumor, n = 48) compared with normal adjacent tissues (Non-tumor, n = 48) (Figure 1F, Table S2). However, hsa_circ_0022460 was not consistently significantly dysregulated in GC cell lines (Figure S1C,D). Therefore, hsa_circ_0022462, named circ*MTA2*, was selected for further investigation of its role in GC.

Next, the total RNA was digested with or without RNAse R to confirm the circular characteristics of circ*MTA2*. As shown in Figure 1G, circ*MTA2* was significantly resistant to RNase R digestion related to the linear form of *MTA2* mRNA. Moreover, circ*MTA2* was predominately localized in the cytoplasm (Figure 1H,I). These results illustrated that circ*MTA2* is upregulated in the GC tissue and cells and may serve as a prognosis marker.

### 2.2. CircMTA2 Promotes the Growth and Aggressiveness of GC

**Figure 2:**
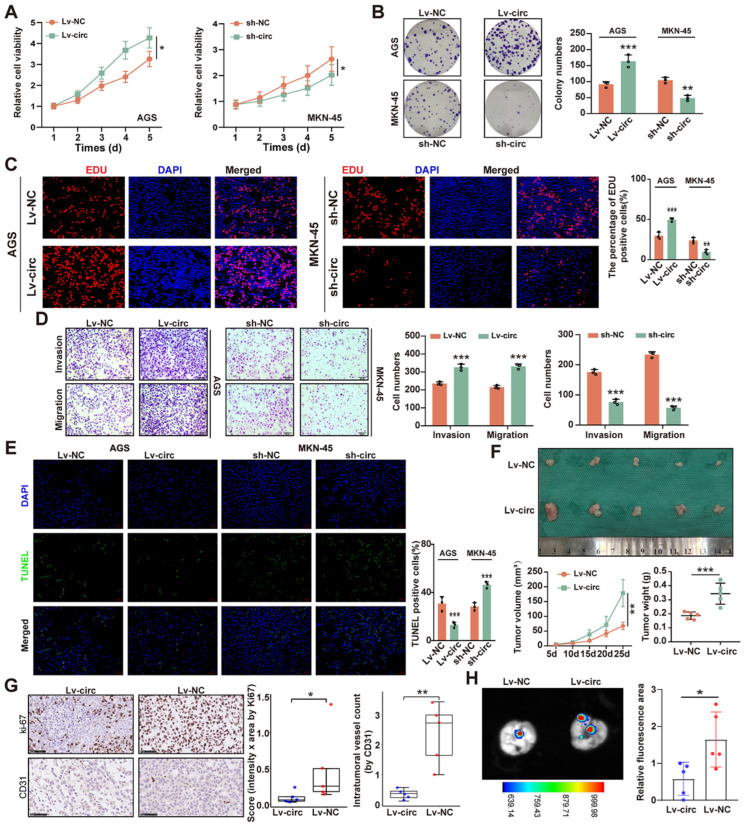
Circ*MTA2* promotes the progression of GC cells. (**A**–**D**) The CCK8 (**A**), colony formation (**B**), EdU incorporation; Scale: 20× (**C**), and transwell assays; Scale: 20× (**D**) show the cell proliferation/invasion/migration status of GC cells stably transfected with empty vector (LV-NC/sh-NC), LV-circ*MTA2* (sh-circ*MTA2*). (**E**) The TUNEL staining indicates the cell apoptosis of GC cells stably transfected with LV-NC/sh-NC and LV-circ*MTA2* (sh-circ*MTA2*); Scale: 20×. (**F**) Representative images, growth curve, and weight at the end points of xenografts formed by subcutaneous xenograft of MKN45 cells stably transfected with LV-NC or LV-circ*MTA2* into the dorsal flanks of nude mice (n = 5 for each group). (**G**) Representative images (left panel) and quantification (right panel) of immunohistochemical staining revealing the expression of Ki-67 and CD31 within xenografts formed by subcutaneous injection of MKN45 cells stably transfected with LV-NC or LV-circ*MTA2*. The scale bar represents 50 µm. (**H**) Fluorescence images of mouse lung metastasis model formed by subcutaneous injection of MKN45 cells stably transfected with LV-NC or lv-circ*MTA2*. All data from three independent experiments shown as mean ± SD. * *p* < 0.05; ** *p* < 0.01; *** *p* < 0.001.

To investigate the functional importance of circ*MTA2* further, we constructed the circ*MTA2* overexpression vector in AGS cells and the circ*MTA2* knockdown vector in MKN-45 cells (Figure S1E). The CCK-8 assay confirmed that overexpression of circ*MTA2* markedly promoted the viability of AGS and MKN-45 cells; however, circ*MTA2* down-regulation markedly inhibited cell viability (Figure 2A). Moreover, circ*MTA2* overexpression significantly increased the colony formation ability of AGS and MKN-45 cells, and circ*MTA2* knockdown showed the opposite result (Figure 2B). Consistently, circ*MTA2* overexpression significantly increased the percentage of EdU-positive nuclei (red) compared to the control in AGS and MKN-45 cells, while circ*MTA2* knockdown had the opposite effect (Figure 2C). Subsequently, we performed a transwell assay upon overexpression or knockdown of circ*MTA2* in AGS and MKN-45 cells. As shown in Figure 2D, stable overexpression of circ*MTA2* enhanced the migration and invasion of GC cells, whereas circ*MTA2* knockdown exerted an inhibitory effect. In addition, the apoptosis rate of GC cells was inhibited by overexpression of circ*MTA2*, whereas it was promoted by knockdown of circ*MTA2* (Figure 2E).

To explore the effect of circ*MTA2* on the growth and metastasis of GC cells in vivo, MKN-45 stable overexpression cells were subcutaneously injected into nude mice. Importantly, overexpression of circ*MTA2* significantly increased tumor growth and volume compared to the control group at 25 days post-injection (Figure 2F). Immunohistochemical (IHC) assay showed that Ki-67 and CD31 positive cell numbers were increased in the circ*MTA2* overexpression group (Figure 2G). Additionally, the lung metastasis images indicated that overexpression of circ*MTA2* promoted tumor metastasis in vivo (Figure 2H). Overall, these findings suggest that circ*MTA2* acts as an oncogene and promotes the progression of GC.

### 2.3. CircMTA2 Stabilities MTA2 Protein by Inhibiting Its Ubiquitination

**Figure 3:**
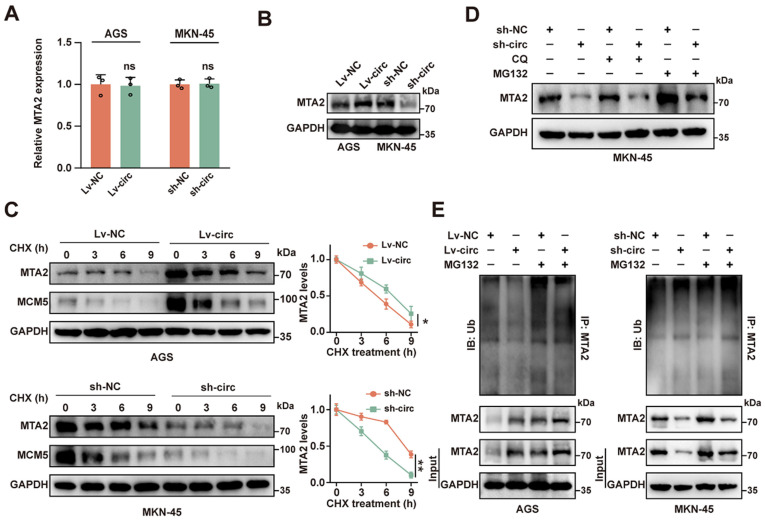
Circ*MTA2* regulates *MTA2* expression by controlling its ubiquitination. (**A**,**B**) qRT-PCR assay (**A**) and Western blot (**B**) analysis indicating the expression of *MTA2* mRNA in GC cells stably transfected with LV-NC/sh-NC and LV-circ*MTA2* (sh-circ*MTA2*). (**C**) CHX chase analysis showing the half-life of *MTA2* protein in GC cells stably transfected with LV-NC/sh-NC and LV-circ*MTA2* (sh-circ*MTA2*). (**D**) Western blot analysis indicated the expression of *MTA2* in MKN-45 cells stably transfected with sh-NC or sh-circ*MTA2* and treated with MG132 or CQ. (**E**) Ubiquitination assay indicating the ubiquitination level of *MTA2* in AGS and MKN-45 cells stably transfected with LV-NC/sh-NC and LV-circ*MTA2* (sh-circ*MTA2*). All data from three independent experiments shown as mean ± SD. ns *p* > 0.05, * *p* < 0.05; ** *p* < 0.01.

To further understand the regulatory relationship between circ*MTA2* and *MTA2*, we examined the changes in *MTA2* expression with circ*MTA2* overexpression and knockdown. Interestingly, circ*MTA2* did not affect the mRNA level of *MTA2* but positively affected *MTA2* protein levels in GC cells (Figure 3A,B). Therefore, circ*MTA2* may modulate the expression level of *MTA2* at the post-transcriptional level by regulating the degradation of *MTA2* protein.

To examine whether circ*MTA2* regulates the stability of *MTA2*, we treated GC cells with cycloheximide (CHX) to inhibit protein synthesis. The results showed that *MTA2* protein was degraded in a time-dependent manner (Figure 3C). Moreover, circ*MTA2* overexpression significantly increased the stability and half-life of *MTA2* in AGS cells, whereas circ*MTA2* knockdown significantly destabilized the *MTA2* protein in MKN-45 cells (Figure 3C). Previous studies have shown that protein degradation is carried out through the autophagolysosome pathway and ubiquitin-proteasome pathway [16]. Subsequently, the two pathway inhibitors, chloroquine diphosphate (CQ) and MG132 were used to treat the GC cells, respectively. As shown in Figure 3D, treatment with MG132 could block the reduction in *MTA2* protein levels caused by circ*MTA2* knockdown, suggesting that circ*MTA2* mainly modulated the ubiquitination pathway of *MTA2*. Notably, overexpression of circ*MTA2* significantly inhibited *MTA2* ubiquitination, whereas knockdown of circ*MTA2* promoted *MTA2* ubiquitination (Figure 3E). Taken together, these results suggested that circ*MTA2* promoted the expression and stability of *MTA2* by inhibiting its ubiquitination.

### 2.4. CircMTA2 Promotes Growth and Aggressiveness of GC by Upregulating MTA2 Expression

**Figure 4:**
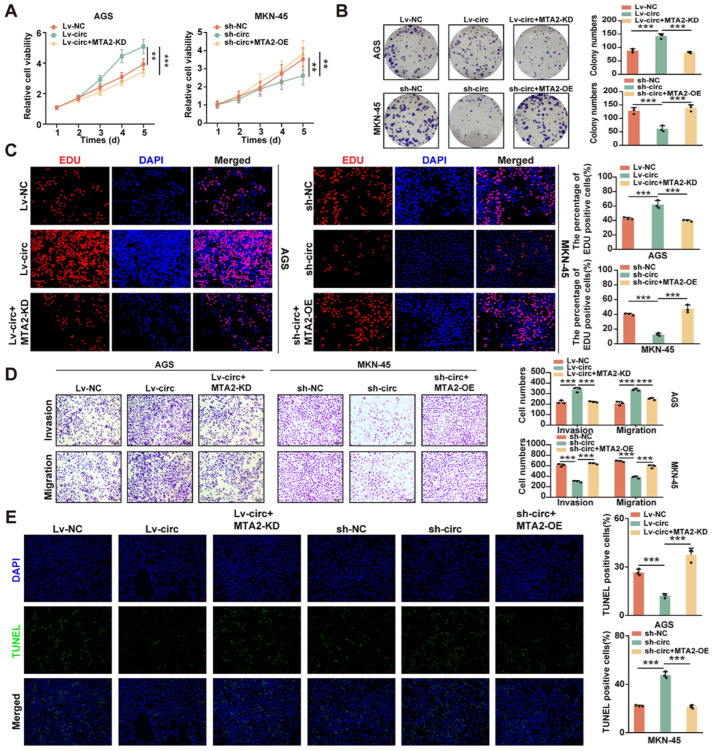
Circ*MTA2* promotes the progression of GC cells by upregulating *MTA2* expression. (**A**–**D**) The CCK-8 (**A**), colony formation (**B**), EdU incorporation; Scale: 20× (**C**), and transwell; Scale: 20× (**D**) assays showing the cell proliferation/invasion/migration status of GC cells stably transfected with LV-NC/sh-NC and LV-circ*MTA2* (sh-circ*MTA2*) plus *MTA2* knockdown/overexpression (*MTA2*-KD/*MTA2*-OE) plasmid. (**E**) The TUNEL staining indicated the cell apoptosis of GC cells stably transfected with LV-NC/sh-NC and LV-circ*MTA2* (sh-circ*MTA2*) plus *MTA2*-KD/*MTA2*-OE plasmid; Scale: 20×. All data from three independent experiments shown as mean ± SD. ** *p* < 0.01; *** *p* < 0.001.

To explore whether circ*MTA2*-mediated *MTA2* expression affects the biological behavior of GC cells, we first performed rescue experiments in AGS cells and MKN-45 cells. Results of CCK-8, colony-formation, and EdU incorporation assays revealed that *MTA2* knockdown significantly abolished the enhancing effect of circ*MTA2* on cell proliferation of AGS cells, while *MTA2* overexpression restored the inhibitory effect of circ*MTA2* knockdown on cell proliferation of MKN-45 cells (Figure 4A–C). Likewise, transwell assays showed that circ*MTA2* overexpression remarkably enhanced cell migration and invasion capacities of AGS cells; however, the promoting effect induced by circ*MTA2* was significantly abrogated by *MTA2* knockdown. The inhibitory effect of circ*MTA2* knockdown on cell migration and invasion was also remarkably rescued by *MTA2* overexpression in MKN-45 cells (Figure 4D). Moreover, the TUNEL assays also explained that circ*MTA2* overexpression suppressed the apoptosis of AGS cells, and *MTA2* knockdown abolished the inhibitory effect of circ*MTA2* on cell apoptosis. These results revealed that Circ*MTA2* promotes the growth and aggressiveness of GC by upregulating *MTA2* expression.

In addition, the promoting effect of circ*MTA2* knockdown on cell apoptosis was remarkably blocked by *MTA2* overexpression in MKN-45 cells (Figure 4E). These data indicated that circ*MTA2* promoted tumor development in GC via enhancing *MTA2* expression.

### 2.5. CircMTA2 Promotes MTA2 Expression via Interacting with UCHL3 in GC Cells

**Figure 5:**
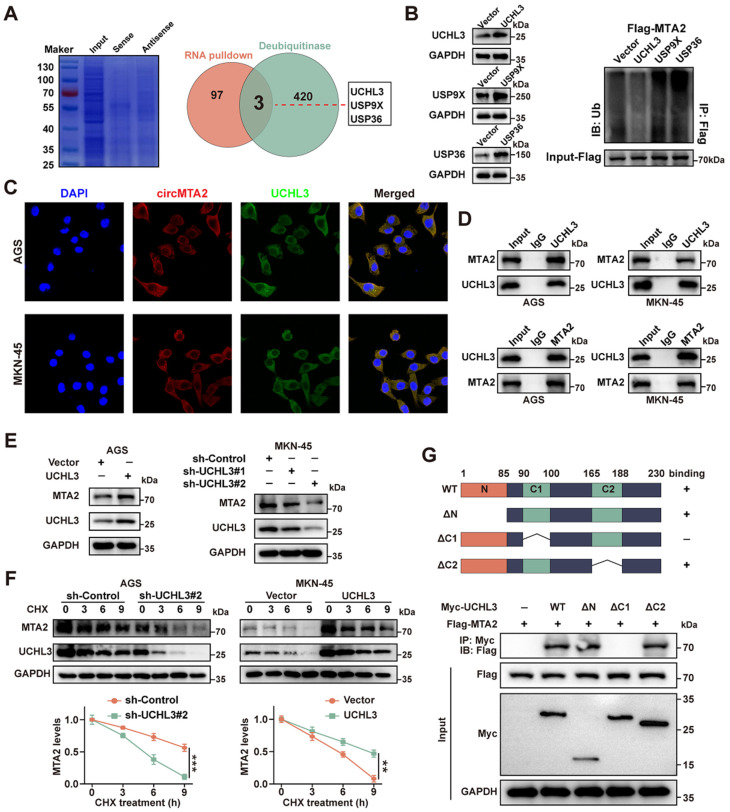
UCHL3 directly combines with *MTA2* in GC cells. (**A**) Coomassie bright blue staining (left panel), Venn diagram (right panel) indicating the differential proteins pulled down by circ*MTA2*, their over-lapping analysis with established deubiquitinase dataset. (**B**) Results of the ubiquitination level of *MTA2* in MKN-45 cells, which were co-transfected with Flag-*MTA2*, HA-Ub, and one of the three potential DUBs. (**C**) Immunofluorescence analysis showing the localization of circ*MTA2* and UCHL3 in GC cells; Scale: 100×. (**D**) Co-IP analysis indicates the interaction between UCHL3 and *MTA2* in GC cells. (**E**) Western blot analysis indicating the expression of *MTA2* in GC cells as indicated. (**F**) CHX chase analysis showing the half-life of *MTA2* protein in GC cells as indicated. (**G**) MKN-45 cells were co-transfected with Myc-UCHL3 and Flag-*MTA2* wildtype or Flag-*MTA2* mutants and harvested for IP analysis. All data from three independent experiments shown as mean ± SD. ** *p* < 0.01; *** *p* < 0.001.

The abovementioned results showed that circ*MTA2* can regulate the expression level and the ubiquitination level of *MTA2*, but circRNA itself does not have the catalytic effect of ubiquitination enzymes; thus, we speculate that a deubiquitination enzyme may play a role. Next, we used circ*MTA2* probes to pull down proteins that might bind to circ*MTA2* and perform mass spectrometry. The results identified three potential circ*MTA2*-interacting deubiquitinating enzymes (DUBs) (UCHL3, UXP9X, and USP36) by overlapping the top 100 detected proteins with the known DUBs list (Figure 5A). Importantly, the ubiquitination level of *MTA2* in AGS cells was significantly affected by UCHL3 rather than UXP9X or USP36 (Figure 5B).

In addition, circ*MTA2* and UCHL3 were co-localized predominantly in the cytoplasm of AGS and MKN-45 cells (Figure 5C). Moreover, the Co-IP assays indicated that UCHL3 and *MTA2* could bind to each other in AGS and MKN-45 cells (Figure 5D). Subsequently, AGS and MKN-45 cells were transfected with overexpression plasmids or two shRNA of UCHL3. The Western blot results showed that the expression level of *MTA2* protein was positively correlated with UCHL3 protein in AGS and MKN-45 cells (Figure 5E).

To investigate further whether the stability of the *MTA2* protein was regulated by UCHL3, we treated the stable UCHL3 knockdown and overexpression GC cells with CHX. Knockdown of UCHL3 in AGS and MKN-45 cells could further decrease *MTA2* expression under CHX treatment, while UCHL3 overexpression partially mitigated *MTA2* decline (Figure 5F). In vitro binding assays indicated that the C1 box domain (90–100 amino acids), but not other domains, of UCHL3 protein specifically bound to *MTA2* (Figure 5G).

**Figure 6:**
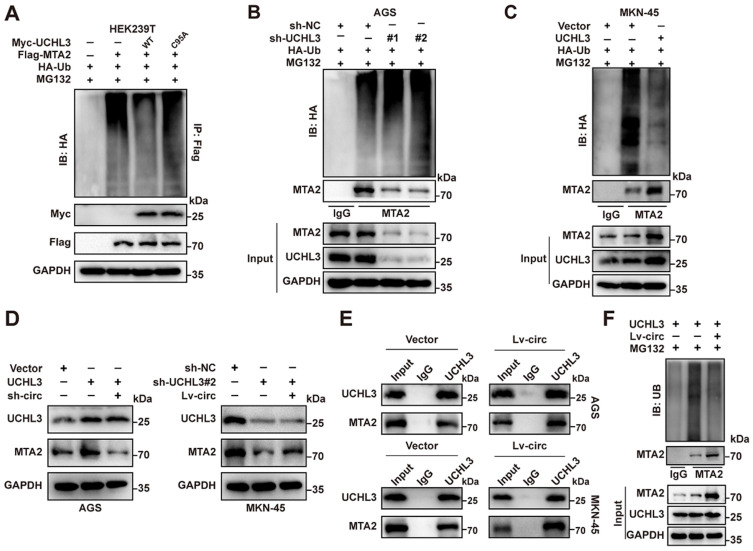
Circ*MTA2* promotes *MTA2* expression via interacting with UCHL3 in GC cells. (**A**) Ubiquitination assays of endogenous *MTA2* in the lysates from HEK239T cells co-transfected with Flag-*MTA2* and Myc-UCHL3 wildtype or Myc-UCHL3 alanine residue mutants. (**B**) Ubiquitination assays of endogenous *MTA2* in the lysates from AGS cells stably transfected as indicated. (**C**) Ubiquitination assays of endogenous *MTA2* in the lysates from MKN-45 cells were stably transfected with an empty vector or a UCHL3 plasmid. (**D**) Western blot analysis indicating the expression of *MTA2* in GC cells stably transfected as indicated. (**E**) Co-IP analysis indicates the interaction between UCHL3 and *MTA2* in GC cells stably transfected with empty vector or LV-circ*MTA2*. (**F**) Ubiquitination assays of endogenous *MTA2* in the lysates from UCHL3 overexpression AGS cells transfected with empty vector, or LV-circ*MTA2*. All data from three independent experiments shown as mean ± SD.

Subsequently, we mutated C95 to alanine (C95A) in UCHL3 (Ubiquitination catalyzed disable mutant). As expected, the mutation of C95A could not catalyze the deubiquitination of *MTA2* protein (Figure 6A). According to the Co-IP results, the knockdown of UCHL3 significantly enhanced the ubiquitination level of the *MTA2* protein and attenuated its binding to *MTA2*, whereas UCHL3 overexpression had the opposite effect (Figure 6B,C). Next, rescue experiments were performed to explore whether circ*MTA2* regulated *MTA2* expression via interacting with UCHL3. As shown in Figure 6D, circ*MTA2* knockdown significantly weakened the enhancement effect of UCHL3 overexpression on *MTA2* protein expression in AGS cells, while circ*MTA2* overexpression yielded the opposite effect in MKN-45 cells. Moreover, overexpression of circ*MTA2* significantly enhanced the interaction between UCHL3 and *MTA2* (Figure 6E) and increased the ubiquitination level of *MTA2* (Figure 6F). Therefore, these findings indicated that circ*MTA2* regulates *MTA2* ubiquitination via interacting with UCHL3.

### 2.6. Exosomal circMTA2 Derived from GC Cells Enhances Cancer Cell Progression

**Figure 7:**
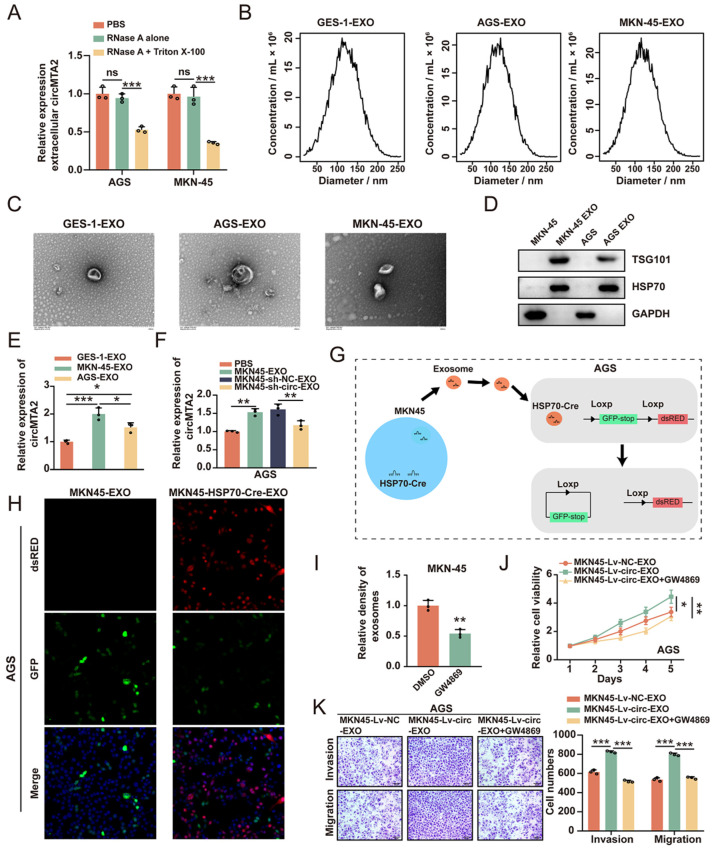
Circ*MTA2* promotes GC cell progression via incorporation into exosomes. (**A**) qRT-PCR analysis indicates the expression of circ*MTA2* in the GC cells medium administered with PBS control, RNase A alone, or RNase A + Triton X-100. (**B**) NTA analysis showing the size range of exosomes isolated from the supernatant of the culture medium of GES-1, GC cells. (**C**) Transmission electron microscopy showing the morphology of exosomes isolated from GES-1 and GC cells. (**D**) Western blot analysis indicates the expression of exosome markers in exosomes isolated from GES-1 and GC cells. (**E**) qRT-PCR analysis indicating the expression of circ*MTA2* in exosomes isolated from GES-1 and GC cells. (**F**) qRT-PCR analysis indicates the expression of circ*MTA2* in exosomes isolated from MKN-45 cells stably transfected with sh-NC or sh-circ*MTA2*. (**G**) The uptake diagram of the exosome. (**H**) Immunofluorescence analysis showing the internalization of fluorescently labeled exosomes in MKN-45 cells; Scale: 20×. (**I**) The density of exosomes derived from AGS cells administered with DMSO control or GW-4869. (**J**,**K**) CCK-8 (**J**) and transwell (**K**) assays indicating the cell proliferation of AGS cells were treated with exosomes derived from circ*MTA2* overexpression MKN45 cells pretreated with DMSO control or GW-4869; Scale: 20×. All data from three independent experiments shown as mean ± SD. ns *p* > 0.05, * *p* < 0.05; ** *p* < 0.01; *** *p* < 0.001.

Mounting evidence has shown that circRNAs could be incorporated into exosomes and thus exert vital functions in intercellular communication [17,18]. Next, we explored whether exosomal circ*MTA2* is critically involved in GC cell progression. First, we observed that the expression level of circ*MTA2* in GC cells medium was remarkably decreased in response to both RNase A and Triton-100 treatment, while there was no change when treated with RNase A alone (Figure 7A). Thus, circ*MTA2* was mainly transferred by exosomes rather than released directly. Subsequently, the exosomes isolated from GES-1, AGS, and MKN-45 were detected by NTA and transmission electron microscopy analysis. These results indicated that the exosomes were approximately 30–200 nm in diameter (Figure 7B,C), and the exosomes isolated from GC cells were enriched with exosomal markers (TSG101 and HSP70) (Figure 7D). In addition, the exosomal circ*MTA2* was significantly more abundant in GC cells than in GES-1 cells (Figure 7E). Moreover, the expression of circ*MTA2* in recipient AGS cells was significantly increased after co-incubation with exosomes from MKN45 cells, while this effect was attenuated when co-cultured with exosomes from circ*MTA2*-knockdown MKN45 cells (Figure 7F). Notably, fluorescence microscopy revealed that exosomes isolated from MKN45-HSP70-Cre cells were co-culture with the AGS-LoxP-eGFP-Stop-LoxP-DsRed, the exosomes were taken up by these AGS cells and successfully converted the green signal to the red signal (Figure 7G,H). To further confirm that exosomal circ*MTA2* played a deterministic role in GC cell progression, we first suppressed the production of exosomes in MKN-45 cells by per-treating with GW4869 (Figure 7I). Subsequently, we isolated exosomes from MKN-45 cells that were transfected with the circ*MTA2*/NC vector (circ*MTA2*/NC-EXO) and found that direct incubation with circ*MTA2*-EXO dramatically enhanced the proliferation invasion and migration of GC cells, while this affection could be blocked by administrating GW4869 (Figure 7J,K). Therefore, these data suggest that exosomal circ*MTA2* derived from GC cells enhances cancer cell progression.

## 3. Materials and Methods

### 3.1. Cell Lines and Treatment

The GC cell lines MKN45 and AGS were bought from Procell (Wuhan, China). SGC7901 and BGC823 were bought from Beyotime (Shanghai, China). MGC803 and the gastric mucosal epithelial cell line GES-1 were purchased from iCell Bioscience (Shanghai, China). All cells were maintained under standard culture conditions using DMEM containing 10% FBS at 37 °C and 5% CO_2_. All cells were checked for mycoplasma by a PCR-based method as well as 4′,6-diamidino-2-phenylindole (DAPI) staining to ensure the absence of contamination.

Cells were seeded in 6-well plates at a density of 2 × 10^5^ per well and cultured in a 37 °C incubator overnight. The shRNA plasmid (GenePharma, Suzhou, China) was transfected into the cells using Lipofectamine 3000 (Invitrogen, Los Angeles, CA, USA) in a serum-free medium. Cells were changed to complete medium at 6 h after transfection and cultured for another 30 h. Stable circ*MTA2* overexpression cell lines were established using lentivirus preparations infection and puromycin (Biosharp, Beijing, China) selection. The target sequences of shRNAs and the overexpression plasmid are provided in Supplementary Table S1.

### 3.2. Patient Tissue Samples

The 48 GC tissues and matched adjacent normal tissues were collected from Union Hospital of Tongji Medical College. Our study was approved by the Ethical Committee of Tongji Medical College for using tissue specimens for research purposes (approval number: 2018-S377). Prior informed consent was obtained from all patients.

### 3.3. Fluorescence In Situ Hybridization (FISH)

Cells were seeded on the slides and fixed with 4% paraformaldehyde. Hybridization was performed with a specific probe for the circ*MTA2* junction site using a Fluorescent in Situ Hybridization Kit (GenePharma, Shanghai, China). Cell nuclei were counterstained with DAPI.

### 3.4. RNA Extraction and Quantitative Real-Time PCR (qRT-PCR)

Total RNA was isolated from tissue samples and cells using Trizol reagent (Invitrogen, Los Angeles, CA, USA) according to the manufacturer’s procedures. qRT-PCR was performed using the SYBR Green PCR kit (Applied TaKaRa, Otsu, Japan) on the ABI 7300 Fast Real-Time PCR System (Applied Biosystems, Foster City, CA, USA). The target genes were normalized to GAPDH to obtain the relative expression level following the 2^−ΔΔCt^ method. The primer sequences are provided in Supplementary Table S1.

### 3.5. RNase R and Actinomycin D Assays

RNase and actinomycin D assays were used to determine the RNA stability. For the RNase R assay, 2 μg RNA was treated with 3 U/μg RNase R (BioVision, PaloAlto, CA, USA) or diethylpyrocarbonate (DEPC)-treated water (control) at 37 °C for 30 min. Thereafter, RNA was extracted using an RNeasy MinElute Cleaning Kit (Qiagen, Helsinki, Germany) and subjected to qRT-PCR quantification. For the actinomycin D assay, cells were treated with 2 μg/mL actinomycin D (Aladdin, Shanghai, China) to halt RNA synthesis. The remaining RNA in the cells was extracted and subjected to qRT-PCR quantification.

### 3.6. RNA-Protein Immunoprecipitation (RIP) Assay

The Magna RIP Kit (Millipore, Billerica, MA, USA) was taken to conduct RIP assays following the manufacturer’s standard protocols. The co-precipitated RNA resultants were extracted and purified via TRIzol (Invitrogen, Carlsbad, CA, USA) and analyzed by qRT-PCR.

### 3.7. RNA Pulldown Assay

The biotinylated circ*MTA2* probe (5′-3′) and control probe (5′-3′) were designed and synthesized by Ribobio (Guangzhou, China). The RNA-protein Pull Down Kit (Thermofisher, Wilmington, MA, USA) was used to conduct the following procedures according to the standard protocols. Briefly, after incubation of the biotinylated probe with streptavidin magnetic beads for half an hour, the magnetic bead–probe complex was incubated with cellular lysates at 4 °C overnight, and the resultants were eluted for RNA analysis using TRIzol (Invitrogen, Carlsbad, CA, USA) or protein analysis using SDS-PAGE loading buffer (Beyotime, Shanghai, China). The abundance of enriched protein was analyzed using Western blotting.

### 3.8. Western Blotting Analysis

Cells were lysed with RIPA buffer (Beyotime, Shanghai, China) containing protease inhibitors (Roche, Basel, Switzerland). Equal amounts of proteins were separated using SDS-polyacrylamide gel electrophoresis (SDS-PAGE) on a 12% polyacrylamide gel. The proteins were transferred electrophoretically onto 0.22 μm PVDF membranes (Millipore, Billerica, MA, USA), blocked in 5% non-fat milk, and then incubated with primary antibodies (MCM5, 1:2000, 11703-1-AP, Proteintech, Chicago, IL, USA; *MTA2*, 1:800, ab8106, Abcam, Cambridge, England; MCM5, 1:2000, 11703-1-AP, Proteintech; USP9X, 1:5000, 55054-1-AP, Proteintech; USP36, 1:5000, 68165-1-Ig, Proteintech; HSP70, 1:8000, 10995-1-AP, Proteintech; TSG101, 1:4000, 28283-1-AP, Proteintech; UCHL3, 1:2000, 12384-1-AP, Proteintech; GAPDH, 1:50,000, 60004-1-Ig, Proteintech; Flag, 1:5000, 66008-4-Ig, Proteintech; Myc, 1:2000, 16286-1-AP, Proteintech; HA, 1:5000, HRP-81290, Proteintech). After incubation with HRP-linked secondary antibody, the protein bands were visualized using chemiluminescence (Millipore, Shanghai, China). GAPDH was used as the loading control.

### 3.9. CCK-8 Assay

CCK-8 assay was performed to evaluate the proliferative ability of cells. Briefly, 1 × 10^3^ cells in different treatment conditions were seeded into each well of a 96-well plate. A volume of 10 μL CCK-8 reagent (Biosharp, Shanghai, China) was added to each well and incubated for 1 h. After a brief shaking of the plate, the absorbance at 450 nm was assessed using a Varioskan LUX machine (ThermoFisher, Waltham, MA, USA).

### 3.10. Colony Formation Assay

Colony-formation assays were carried out to evaluate the cloning capability of colorectal cancer cells. In brief, the indicated cells were seeded onto 6-well culture plates until visible colonies appeared, and the complete medium was replaced every 4 days. Subsequently, the cell colonies were fixed in 95% methanol for 20 min, stained with 0.1% crystal violet (Beyotime, Nanjing, China) for 15 min, and quantified using ImageJ software (version 1.8.0.112; National Institutes of Health).

### 3.11. 5-Ethynyl-20-Deoxyuridine (EdU) Analysis

Before transfection, cells were seeded on coverslips in 24-well plates and incubated according to standard culture conditions. After 72 h, EdU was added to the culture medium and incubated at 37 °C for 2 h. Then, they were fixed with 4% paraformaldehyde for 15 min at room temperature. After washing off the paraformaldehyde, 0.3% Triton X-100 was added (Invitrogen, Carlsbad, CA, USA) to the plates and incubated for 20 min. Next, Click Additive Solution was added according to the instruction provided by the commercial protocol (BeyoClick™ EdU-555, Shanghai, China), then incubated at room temperature for 30 min, protected in the dark. Finally, the nucleus was stained with Hoechst 33342, and images were taken using a fluorescent microscope (Olympus, Tokyo, Japan).

### 3.12. Terminal Deoxynucleotidyl Transferase (TdT)-Mediated dUTP Biotin Nick End Labeling (TUNEL) Staining

For the apoptosis assay, the TUNEL assay kit (Beyotime, Nanjing, China) was used to stain the cells based on the manufacturer’s manual. Briefly, GC cells were fixed with 4% polyformaldehyde for 30 min, followed by washing with PBS. Next, the TUNEL reaction mixture was added to the cells and incubated 1 h at 37 °C. After washing with PBS and DAPI staining, the cells were examined and imaged under a light microscope (Olympus, Tokyo, Japan).

### 3.13. Transwell Assay

The protocols are the same as in the previous article [15]. Transwell assay was conducted with a transwell chamber (Corning, Shanghai, China). The upper chamber was filled with cells and 200 μL serum-free medium, while the lower chamber was filled with 600 μL medium with 25% FBS. The cells on the lower side of the membrane were fixed and dyed with 0.1% crystal violet after culturing for 24 h.

### 3.14. Xenograft Assay

The animal experiments were approved by the Institutional Animal Care and Use Committee of Tongji Medical College, Huazhong University of Science and Technology, and the xenograft assays were performed as previously described [19].

### 3.15. Immunohistochemical (IHC) Assay

For IHC analyses, 4% paraformaldehyde-fixed tissues were embedded in paraffin and cut into 4 μm-thick sections. The sections were incubated with primary monoclonal antibody against Ki-67 (Abcam, ab15580) and CD31 (Abcam, ab281583), followed by incubation with the secondary antibody for 30 min at room temperature. Finally, the sections were counterstained with hematoxylin and photographed under a TE2000 microscope (Nikon, Tokyo, Japan).

### 3.16. Exosome Isolation and Characterization

First, cells were cultured in exosome-free serum according to the conditions required by the experiment to remove the interference of serum exosomes. The serum was placed in a high-speed centrifuge (100,000× *g*, Hitachi, Tokyo, Japan) at 4 °C for ultracentrifugation for more than 16 h, and then the FBS supernatant was filtered using a filter (0.22 μm, Millipore, Billerica, MA, USA). The final filter was fetal bovine serum without exosomes. MKN45/AGS cells were cultured using the 10% exosomes-free FBS medium under plasmid treatment. The medium was collected after 24–72 h. The following exosome isolation was performed according to the instructions of the kit (Invitrogen, Carlsbad, CA, USA). Briefly, the media was spun (2000× *g*, Hitachi, Tokyo, Japan) for 30 min to remove cells and debris. Then, 0.5 volume equivalents of total exosome isolation reagent were added, and the samples were incubated at 2 °C to 8 °C overnight. Exosomes were centrifuged (10,000× *g*, Hitachi, Tokyo, Japan) at 2 °C to 8 °C for 1 h and resuspended with PBS. Exosome sizes and concentrations were quantified using a qNano, and the morphology was captured using an electron microscope. Exosomes were labeled with PKH26 following the instructions and consequently extracted again from the regent. The plasma exosomes were also extracted as per the manufacturer’s instructions (Invitrogen, Carlsbad, CA, USA). Plasma samples were centrifuged at 2000× *g* (Hitachi, Tokyo, Japan) for 20 min and 10,000× *g* (Hitachi, Tokyo, Japan) for 20 min to remove cells and debris and incubated with PBS for 10 min. Then, a 0.2 volume equivalent of exosome precipitation reagent was added. The exosomes were suspended with PBS after centrifugation (10,000× *g*, Hitachi, Tokyo, Japan) for 5 min at room temperature. For the exosome treatment, exosomes were isolated from 5 × 10^6^ GC cells cultured under the corresponding treatment, and the cells were planted in 12-well plates the day before treatment. A total of 100 μg of exosomes were added to the plates once the cells grew at a confluence of about 70%. After 48 h, the cells were prepared for the following experiment. PKH26 (cat: HY-D1451) and GW4869 (Cat: HY-19363) were purchased from MedChemExpress. PKH26 (10 μM) was incubated with exosomes for 5 min at 37 °C to label exosomes. GW4869 (20 μM) was incubated with cells for 30 min at 37 °C to inhibit the release of exosomes.

## 4. Statistical Analysis

Statistical analyses were performed using GraphPad Prism version 8 for Windows or R 4.0.2. Statistically significant differences were calculated using Student’s *t*-test and one-way ANOVA as needed. *p* < 0.05 was considered significant.

## 5. Discussion

In this study, we aim to verify whether circRNA is involved in the regulation of *MTA2* expression, thereby contributing to the progression of GC. First, we identified that circ*MTA2* (hsa_circ_0022462) was a 300 nt transcript originating from *MTA2* gene exon-4, exon-5, and exon-6. Moreover, circ*MTA2* was significantly upregulated in GC tissues and cells and was associated with GC progression in vitro and in vivo. Of note, we found that circ*MTA2*, as a promoter of GC proliferation and migration, maintains the stability of *MTA2* protein through UCHL3-mediated *MTA2* deubiquitination. Taken together, these findings provide insights into the molecular mechanisms behind GC tumorigenesis.

As a core component in the nucleosome remodeling and deacetylating (NuRD) complex, *MTA2* plays a vital role in global gene expression regulation [13,20]. For instance, *MTA2* promotes carcinogenesis and progression of pancreatic ductal adenocarcinoma by inhibiting the expression of PTEN [21]. Ectopic expression of *MTA2* enhances the metastatic potential of cervical cancer cells via promoting AP1-mediated MMP12 expression [22]. *MTA2* mainly acts as a transcription factor that has been amplified in many types of cancers, including GC [13,14,15,21]. However, the mechanism supporting abnormal *MTA2* expression in GC has not yet been delineated. Recently, circRNAs have emerged as key regulators in gene regulation [7,23]. Our previous study also found that *MTA2* is highly expressed in GC tissues and cells, and *MTA2* inhibition attenuates the progression of GC cells by inhibiting MCM5 expression [15]. Herein, our key findings revealed that circ*MTA2* is a crucial and novel modulator that enhances the stability of *MTA2* protein by inhibiting its ubiquitination (Figure 6). Overexpression of circ*MTA2* was highly associated with tumor progression by upregulating *MTA2* protein levels in GC cells. Of note, circ*MTA2* could interact with UCHL3, which ultimately reduced the ubiquitination degradation of *MTA2*, thus promoting GC progression. Similarly, Zhang et al. found that enhanced degradation of *MTA2* could suppress the metastatic ability of luminal B breast cancer cells [24]. Previous studies have shown that the ubiquitin-proteasome system is an important regulator of protein-targeted degradation involved in a variety of cellular processes [25,26].

The deubiquitinase UCHL3 is emerging as an important regulator in human cancer. UCHL3 increases the stability of the YAP protein in a deubiquitinating activity-dependent manner, thereby promoting the progression and metastasis of anaplastic thyroid cancer [27]. Moreover, UCHL3 in non-small cell lung cancers promotes stem-like characteristics and potent tumorigenic capacity by deubiquitinating the aryl hydrocarbon receptor [28]. Previous studies have reported that circRNA can combine with DUBs to reduce the ubiquitination of downstream target proteins, thereby regulating tumor progression [29]. For instance, circWSB1 promoted the proliferation of breast cancer via competitively binding to deubiquitinase USP10 and leading to the degradation of p53 [29]. In the present study, we identified *MTA2* as a new substrate of UCHL3 in GC. Overexpression of UCHL3 in MKN-45 cells significantly increased the stability and half-life of the *MTA2* protein. Moreover, circ*MTA2* knockdown significantly weakened the enhancement effect of UCHL3 overexpression on *MTA2* protein expression in AGS cells. Therefore, the UCHL3-*MTA2* axis plays a crucial role in circ*MTA2*-mediated *MTA2* stabilization.

Emerging studies have demonstrated that exosome-mediated transfer of circRNAs plays a crucial role in regulating intercellular communication in the tumor microenvironment [30,31]. For instance, Xu et al. have reported that exosomal circSORE derived from hepatocellular carcinoma cells promotes sorafenib resistance by blocking PRP19-mediated YBX1 degradation [32]. Exosomal circSHKBP1 promoted GC cell proliferation, migration, invasion, and angiogenesis by regulating the miR-582-3p/HUR/VEGF pathway and suppressing HSP90 degradation [33].

Likewise, we found that circ*MTA2* was significantly enriched in GC cell-derived exosomes. More importantly, circ*MTA2* overexpression in GC cell-derived exosomes remarkably promoted GC cell proliferation, migration, and invasion. Knockdown of exosomal circ*MTA2* also significantly inhibits GC cell invasion and migration. Thus, exosomal circ*MTA2* secreted by GC cells promotes the progression of GC by upregulating *MTA2* protein expression and targeting exosomal circ*MTA2*, which may be a promising strategy for the treatment of GC patients.

In conclusion, our study first revealed that circ*MTA2* as an oncogene plays a crucial role in mediating the progression of GC through stabilizing *MTA2* by interacting with UHL3. Hence, preventing the circ*MTA2*/UHL3/*MTA2* axis may be a promising therapeutic strategy for GC treatment.

## Data Availability

Data are contained within the article and Supplementary Materials.

## Supplementary Materials

The following supporting information can be downloaded at: https://www.mdpi.com/article/10.3390/ijms25052817/s1.
