# Supplementary material for: CircMTA2 Drives Gastric Cancer Progression through Suppressing MTA2 Degradation via Interacting with UCHL3
Source: Int J Mol Sci. 2024 Feb 29;25(5):2817. doi: 10.3390/ijms25052817 (PMC10932366; doi:10.3390/ijms25052817)
Supplement: Supplementary file 1 [file ijms-25-02817-s001.zip › ijms-2730369-supplementary.pdf]

Figure S1. The expression of Hsa_circ_0022460 in the GC tissue and cells.

(A) Genomic location and sanger sequence validation of hsa_circ_0022460. **(B)** qRT-PCR assay indicating the circularization structure of hsa_circ_0022460. **(C)** Relative hsa_circ_0022460 expression levels in GC and GES-1 cells. **(D)** Relative hsa_circ_0022460 expression levels in GC tissues and adjacent normal tissues. **(E)** qRT-PCR assay indicating the expression of circMTA2 in AGS and MKN-45 cells stably transfected with lv-NC, lv-circMTA2, sh-NC, or sh-circMTA2.

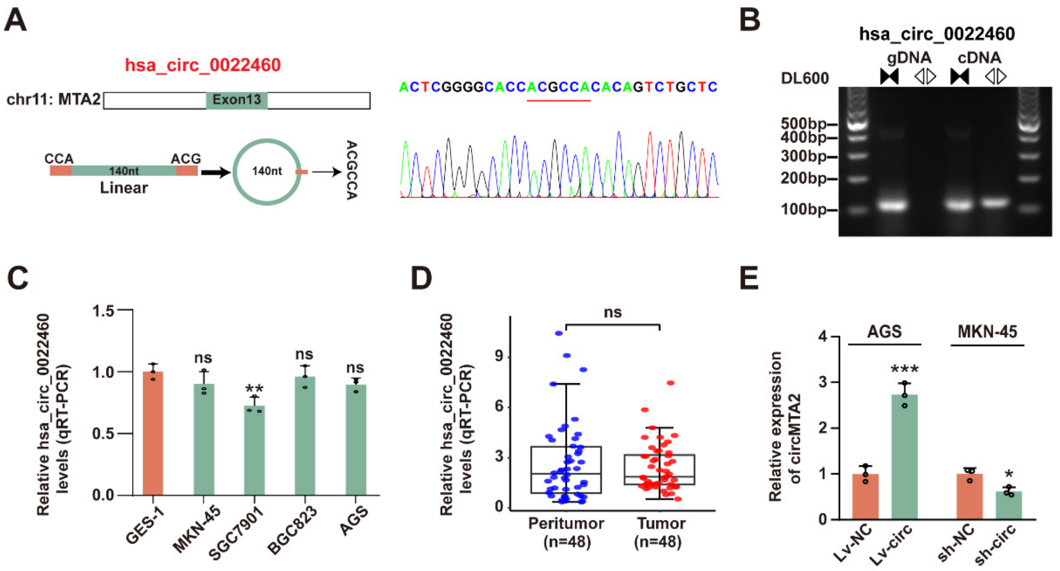

Table S1. All special primers and short hairpin RNA presented in our study.

Primer	Sequence
hsa_circ_0022462	Forward: 5′- AGGGCGAGATTAGAGTTGGT -3′
(Divergent)	Reverse: 5′- TCAAGAGGGTCACACTGCAT -3′
hsa_circ_0022462	Forward: 5′- GAGGAATCAAAGCAGCCAGG-3′
(Convergent)	Reverse: 5′- ACCAACTCTAATCTCGCCCT-3′
hsa_circ_0022460	Forward: 5′- AAGACCCCAACTCAGCTTGA -3′
(Divergent)	Reverse: 5′- CAACAGGAAGCACAGAGGC -3′
hsa_circ_0022460	Forward: 5′- AGTCTGCTCAGTGGTATGCC-3′

(Convergent)	Reverse: 5′- TGAGTTGGGGTCTTCAGTCC-3′
hsa_circ_0022463	Forward: 5′-GTGTTGAAACAATCTCTACTG-3′
(Divergent)	Reverse: 5′-GAACATGTCTGCGTATCTC-3′
hsa_circ_0022458	Forward: 5′- CTTCCTAAGGCCGCCAAGA -3′
(Divergent)	Reverse: 5′- TTCCTCCATCTCATCCCGAC -3′
hsa_circ_0022454	Forward: 5′- TGGAGTTTGAGGCCCCTAAG-3′
(Divergent)	Reverse: 5′- GAGTGATATCTCGGGAGGCA-3′
hsa_circ_0022457	Forward: 5′- CCGCCAAGACTCCATTGAAG-3′
(Divergent)	Reverse: 5′- ACTGGACTATGCTGGCAAGT-3′
hsa_circ_0022461	Forward: 5′- TCAGCAGAAAAGGTTGAAAGC-3′
(Divergent)	Reverse: 5′- ACTGGACTATGCTGGCAAGT-3′
hsa_circ_0022459	Forward: 5′- GCAGGGACCTATTACAGCCA-3′
(Divergent)	Reverse: 5′- TCTGTGAGAGGGTTGTCTGG-3′
hsa_circ_0022456	Forward: 5′- AATCCTGTGGTGTTTGTGGC-3′
(Divergent)	Reverse: 5′- TGAGTTGGGGTCTTCAGTCC-3′
hsa_circ_0096057	Forward: 5′- GCAGGGACCTATTACAGCCA-3′
(Divergent)	Reverse: 5′- CGCTGGTTGTGTAAGGAGAG-3′
hsa_circ_0022455	Forward: 5′- AATCCTGTGGTGTTTGTGGC-3′
(Divergent)	Reverse: 5′- TGTGAGCTTGAACGAATCCC-3′
MTA2	Forward: 5′- CGGGATGAGATGGAGGAAT-3′
	Reverse: 5′- GGTTAGGGTTTGGCTTAGTGTA-3′
GAPDH	Forward: 5′- CGGATTTGGTCGTATTGGG-3′
	Reverse: 5′- TCTCGCTCCTGGAAGATGG-3′
hsa_circ_0022462 (shRNA)	5'CCGGCCTAGTAGAGGGGGAGTTTGAACTCGAGTTCAAACTCCCCCTCTACTAGGTTTTTG 3' (Sense)
	5'AATTCAAAAACCTAGTAGAGGGGGAGTTTGAACTCGAGTTCAAACTCCCCCTCTACTAGG 3' (Anti-sense)
sh-UCHL3 #1	5'CCGGGCATTAGTTCATGTAGATGGGCTCGAGCCCATCTACATGAACTAATGCTTTTTG 3' (Sense)
	5'AATTCAAAAAGCATTAGTTCATGTAGATGGGCTCGAGCCCATCTACATGAACTAATGC 3' (Anti-sense)
sh-UCHL3 #2	5'CCGGGAGGATGCCATAGAAGTTTGCCTCGAGGCAAACTTCTATGGCATCCTCTTTTTG 3' (Sense)
	5'AATTCAAAAAGAGGATGCCATAGAAGTTTGCCTCGAGGCAAACTTCTATGGCATCCTC 3' (Anti-sense)

Table S2. Clinical relevance of the circMTA2/MTA2 axis in GC.

Clinicopathologic parameter	Number	Number of patients(circMTA2)		p value	Number of patients (MTA2)		p value
		Low n=24	High n=24		Low n=24	High n=24	
Age							
<60y	21	12	9	0.682	11	10	0.842
≥60y	27	12	15		13	14	
Gender							
Male	32	17	15	0.129	16	16	0.149
Female	16	7	9		8	8	
Tumor size							
<3cm	17	12	5	0.043*	9	4	0.007*
≥3cm	31	12	19		15	20	
Tumor site							
Proximal	22	10	12	0.881	9	11	0.644
Non-proximal	26	14	12		15	13	
Lymph node metastasis							
N0	16	12	4	0.014*	10	6	0.083
N1-N3	32	12	20		14	18	
TNM stage							
I-II	18	14	4	0.010*	13	5	0.040*
III	30	10	20		11	19	
Blood vessel invasion							
Negative	14	9	5	0.022*	8	6	0.034*
Positive	34	15	19		16	18	

*P<0.05 indicates a significant relationship among the variables.
